# FGFR inhibitors in cholangiocarcinoma: what’s now and what’s next?

**DOI:** 10.1177/1758835920953293

**Published:** 2020-09-16

**Authors:** Anna Saborowski, Ulrich Lehmann, Arndt Vogel

**Affiliations:** Department of Gastroenterology, Hepatology & Endokrinologie, Medizinische Hochschule Hannover, Hannover, Germany; Institute of Pathology, Medizinische Hochschule Hannover, Hannover, Germany; Department of Gastroenterology, Hepatology and Endocrinology, Hannover Medical School, Carl-Neuberg Str. 1, Hannover, 30625, Germany

**Keywords:** biliary tract cancer, genetic rearrangement, next generation sequencing, precision oncology, translational medicine

## Abstract

Patients with intrahepatic cholangiocarcinoma (iCCA) face a highly dismal prognosis, due to late stage diagnosis, the relative chemoresistance of the disease, and an overall limited portfolio of established therapeutic concepts. In recent years, a number of next generation sequencing studies have provided detailed information on the molecular landscape of biliary malignancies, and have laid the groundwork for the evaluation of novel, targeted therapeutic opportunities. Although nearly 40% of patients harbor genetic alterations for which targeted options exist, rapid translation into clinical trials is hampered by the overall low patient numbers. One of the most frequent genetic events in patients with iCCAs are fusions that involve the fibroblast growth factor receptor 2 (*FGFR2*). Impressive results from pivotal phase II studies in pre-treated patients have confirmed that FGFR-inhibitors are a promising therapeutic option for this genetic subgroup, and the rapid pace with which these inhibitors are being clinically developed is clearly justified by the imminent benefit for the patients. However, the success of these agents should not blind us to key challenges that need to be addressed to optimize FGFR-directed therapies in the future. A better understanding of mechanisms that convey primary and secondary resistance will be crucial to improve up-front patient stratification, to prolong the duration of response, and to implement reasonable co-treatment approaches. In this review, we provide background information on the pathobiology of oncogenic FGFR fusions and selected genetic testing strategies, summarize the latest clinical data, and discuss future directions of FGFR-directed therapies in patients with iCCA.

## Cholangiocarcinoma

Cholangiocarcinomas (CCAs) are highly aggressive tumors that display features of biliary differentiation. CCAs can arise either within the liver, termed intrahepatic cholangiocarcinoma (iCCA), or in the perihilar or distal portions of the draining bile ducts (perihilar or distal CCA, respectively). In most countries, CCAs are considered “rare” cancers with incidence rates below 6/100,000. However, reflecting the distribution of different risk factors, and likely also different ethnic backgrounds, CCA incidence ranges from 0.1/100,000 in Australia to more than 110/100,000 in Northeast Thailand.^1,2^

Surgical resection is the only potentially curative option and should be offered to patients that are diagnosed at early stages. However, due to late manifestation of clinical symptoms, most patients present with locally advanced or metastatic disease, and, even after complete resection, the majority experience rapid recurrence. Therefore, palliative treatments are the mainstay of CCA therapy. Unfortunately, CCAs are highly chemotherapy refractory malignancies, with a median overall survival (mOS) of 11–13 months under first-line palliative treatment with gemcitabine and cisplatin.^3,4^ Except for initial data from the United Kingdom (UK) in favor of second-line therapy with 5-FU and oxaliplatin, no established second line therapeutic concepts exist (ABC-06 trial).^5^

In recent years, sequencing studies have addressed the genetic underpinnings of CCA. Overall, CCAs are genetically heterogeneous, and the molecular profiles segregate with the anatomical location (intrahepatic *versus* perihilar or distal CCA), the histological subtype, as well as with the putative pathogenic risk factors, thus adding to the complexity of the disease. Despite the genetic diversity, a recurrent repertoire of driver genes and potentially targetable lesions exists: indeed, several studies suggest that about 40% of patients harbor targetable alterations, indicating precision oncology has the potential to complement existing therapies.^6–10^ Recently, the first randomized phase III study that investigated a precision oncology-based concept in a genetically selected CCA patient cohort was completed and reported positive data for the IDH-inhibitor ivosidenib in IDH1 mutant patients.^11^

An emerging “class” of drug targets in oncology are fusion oncogenes, and, specifically in CCA, fusions that involve the fibroblast growth factor receptor 2 (FGFR2). These fusions are detected in 10–15% of patients with iCCA. Of note, they are found nearly exclusively in intrahepatic, but not in perihilar or extrahepatic CCA, or hepatocellular carcinoma.^9,10,12^ Fusions that involve other members of the FGFR family are rare in biliary tract cancers, with an incidence below 0.5%.^13^ Although there is initial evidence that FGFR2 genetic alterations occur more frequently in younger patients and are associated with a more indolent disease progression,^13^ it remains enigmatic whether FGFR2 fusion positive patients represent a distinct prognostic subgroup.

## FGFR signaling

The fibroblast growth factor receptor (FGFR) family consists of four subtypes of transmembrane tyrosine kinase receptors, FGFR1–4.^14^ In addition, a structurally related protein that lacks the tyrosine kinase domain – FGFR5 – has recently been suggested to function as a co-receptor for FGFR1.^15^ While physiological FGFR signaling plays an important role in several cellular processes such as proliferation, survival, migration, and angiogenesis, dysregulated FGFR activity can set the stage for malignant transformation. In addition, increased FGF/FGFR signaling has been described as a secondary resistance mechanism to targeted therapies.^16^

The binding of FGF ligands to their respective receptors results in receptor dimerization and subsequent transphosphorylation of the tyrosine kinase domains.^17^ Key downstream substrates include PLC-y-mediated activation of PKC, as well as pFRS2-induced activation of PI3K and MAPK, but, depending on the (cellular) context, several other pathways may as well be affected, such as c-JUN and STAT-signaling. Activation of FGFR2 is subject to especially complex control mechanisms, that involve the receptor being “held” in a dimeric state by binding of a Grb2 dimer, leading on the one hand to a partial phosphorylation of the receptor, but on the other hand inhibiting the phosphorylation of additional residues that are crucial for recruitment of downstream signaling proteins. Only in the presence of FGF ligands, upregulation of the kinase activity releases Grb2 through phosphorylation, permitting active signal transduction through FGFR2.^18–20^

FGFR2 fusions typically result from chromosomal events that lead to an *in frame* fusion between the 5′ portion of the *FGFR2* gene, and a partner gene. FGFR2 is located on chromosome 10, and around 50% of FGFR2-fusions evolve through intrachromosomal events.^21^ On a structural level, the *FGFR2* portion of the fusion gene retains the extracellular domain, as well as the kinase-domain, whereas the fusion partner contributes a dimerization signal, leading to constitutive, ligand-independent, pathway activation.

In light of the therapeutic relevance, which we will discuss later in this review, a reliable identification of fusion positive iCCA patients is crucial. Therefore, it is important to be aware that not all diagnostic strategies are equally suited, and that detection of FGFR2 fusions as well as other fusion oncogenes is highly dependent on the selection of an appropriate testing strategy. A diagnostic advantage is that the location of the breakpoint in the FGFR2 gene appears to be nearly universal within intron 17 or exon 18 (INCYTE, personal communication A. Vogel). However, with respect to potential partner genes, more than 150 fusion partners have been described until now, albeit with variable frequency.

## Methodological considerations for the detection of therapeutically relevant fusion transcripts

Promoter activation or loss of negative regulatory elements can result in highly active transcription of oncogenic fusion DNA and frequently leads to overexpression of the corresponding fusion proteins. This overexpression can be detected by traditional immunohistochemistry and serve as a surrogate marker for the presence of a fusion gene. Immunohistochemistry is readily available in most laboratories, relatively cost-effective, requires only a single tissue section, and has a very short turnaround time. However, specificity, reproducibility, and comparability of staining results between different laboratories are often difficult issues. In addition, robust and highly specific antibodies are not necessarily available for all targets of interests. The fusion partner might also compromise the staining quality by interfering with proper binding to the target protein. In CCA, wildtype FGFR2 is frequently expressed on tumor cells, as well as on normal cholangiocytes, thereby preventing reliable detection of the fusion protein. Therefore, although immunohistochemistry is under many circumstances a useful and easily implemented screening tool, it is not suitable for the detection of FGFR2 fusions in iCCA patients.

A second cost-effective, sensitive and fast approach to detect specific fusion transcripts is conventional multiplex polymerase chain reaction (PCR). However, with a growing number of fusion partners, to screen for FGFR2 fusions would require designing and using a high number of different primer pairs in a single assay. Importantly, this approach would not be comprehensive as it would fail to identify patients harboring novel FGFR2 fusions. For instance, it would have missed approximately 50% of the FGFR2 fusions seen in the recent FIGHT-202 study.^22^

Fluorescence *in situ* hybridization (FISH) is also a well-established and widely used technique that is available in most laboratories for the analysis of chromosomal alterations. Fusions can be conveniently detected by an adaptation of the FISH technique, called break-apart FISH, which uses two fluorescent (e.g., red and green) DNA probes that are designed to target sequences flanking the gene of interest. Due to the proximity of the probes, a yellow signal is observed under the microscope, whereas, in the event of a chromosomal rearrangement, the probes become separated, resulting in distinct red and green signals. Therefore, break-apart FISH is suitable to detect both known and novel gene fusions, but does not allow identification of the fusion partner. In addition, this method is generally limited to one genetic alteration per slide, and currently no validated test to detect FGFR2 fusions *via* break-apart FISH is widely available.

The most comprehensive protocols for the identification of fusion transcripts are based on next generation sequencing (NGS) technologies, and gene fusions can be detected by sequencing genomic DNA or RNA (*via* cDNA). NGS-based fusion detection employs two distinct approaches: the regions of interest can either be enriched for the sequencing reaction by so-called hybrid capture probes or by amplification using flanking primers (amplicon-approach).

**Figure 1.**
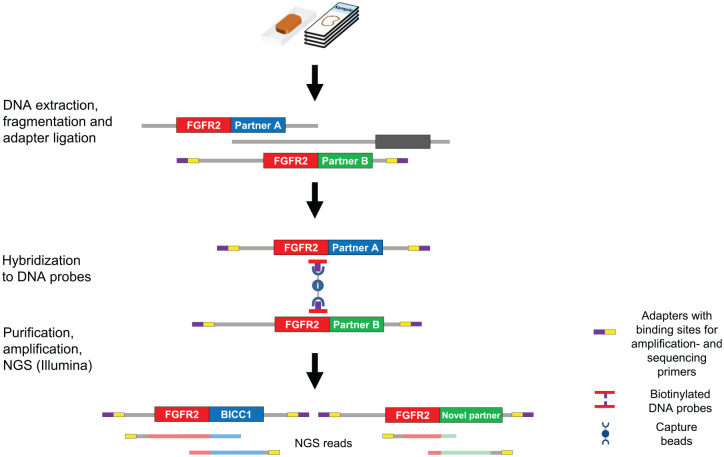
Hybrid capture-based method to generate target-enriched DNA libraries from FFPE tumor tissue (e.g., FoundationOneCDx®) adapted from Jennings et al.^25^

Some of the clinically most frequently used panel sequencing assays employ a hybrid capture-based method to generate target-enriched DNA libraries from FFPE tumor tissue (e.g. FoundationOneCDx®) (Figure 1).^23^ Chromosomal translocations resulting in FGFR2 fusions nearly always occur in intron 17 or exon 18, which allows the design of highly specific hybrid capture probes close to the fusion breakpoints. After isolation, tumor DNA is randomly sheared into smaller fragments, sequencing adapters are attached, and the capture probes hybridize to the target DNA sequences. Captured DNA is then purified, amplified and sequenced, thereby allowing for the identification of FGFR2 fusions without prior knowledge of the fusion partner identity. Of note, whereas highly reliable for the detection of FGFR2 fusions, the sensitivity of hybrid capture assays may vary depending on the fusion; for instance, detection of some NTRK3 fusions can be technically challenging, since the breakpoints occur in intronic regions with reduced sequence complexity (repetitive sequences) or high proportion of the DNA bases adenine and thymine (“AT-rich”) that are so large that they cannot be faithfully covered by capture probes.^24^

Other NGS-based approaches that are applied frequently in daily clinical practice use DNA and cDNA from FFPE tissues that are converted into amplicon libraries, which target the variants and gene fusions of interest (e.g., ONCOMINE assays).^26^ Of note, amplicon-based approaches can detect only known fusion transcripts for which validated primer pairs have been designed and included in the panel, and thus cannot be considered an unbiased approach for detection of novel or less common rearrangements (including several FGFR2 fusions). However, the advantage of the amplicon approach is its superior sensitivity compared with the hybrid capture or the anchored multiplex PCR protocols described in the following.

**Figure 2.**
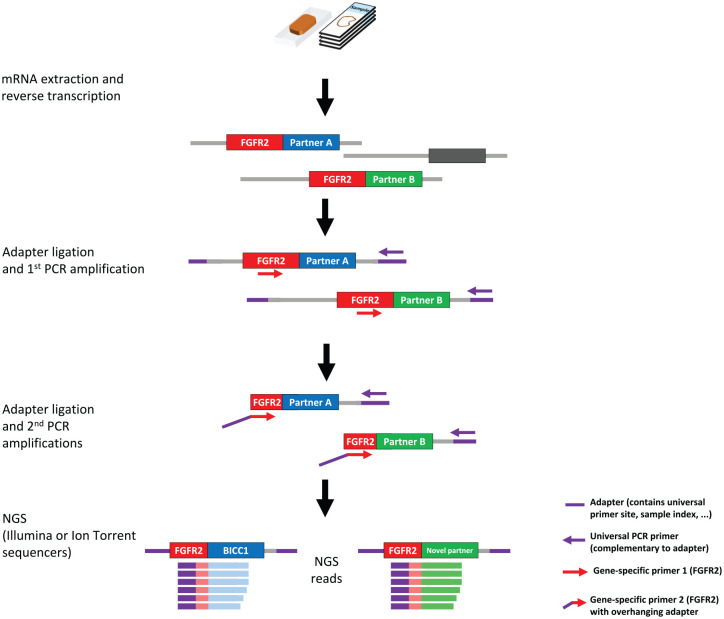
Amplicon-based method to generate target-enriched cDNA libraries from FFPE tumor tissue mRNA (e.g. Archer FusionPlex®) adapted from Jennings et al.^25^

Archer FusionPlex® is a third NGS-based approach and utilizes target-enriched cDNA libraries (Figure 2). Unlike conventional multiplex PCR that uses pairs of gene-specific primers, the assay enables detection of all fusions associated with the genes of interest in a single sequencing assay, even without prior knowledge of fusion partners or breakpoints. Adaptors that harbor a universal primer binding site are ligated to cDNA fragments, and targets are amplified using a gene-specific and one universal primer. The exon level detection by the Archer assay and the amplicon-based assays targeting cDNA provide direct evidence of expression of a functional in-frame fusion transcript, and allow easier primer/probe design because low complexity or repetitive sequences are less frequent in coding sequences within exons.

## Clinical studies with FGFR inhibitors in CCA

Considering the relatively small benefit of first-line chemotherapy, the lack of efficient second-line regimens, and thus far rather disappointing results for immune oncology (IO) concepts in iCCA patients, FGFR2 fusions are promising targets for precision oncology: indeed, thus far, a number of phase II studies have been published that report a clinically meaningful benefit for FGFR2-directed therapies. Two larger single-arm phase II trials reported strikingly similar results in fusion-positive patients treated with different oral ATP-competitive FGFR inhibitors: objective responses were 31% (22/71) with an estimated mPFS of 5.8 months for infigratinib [ClinicalTrials.gov identifier: NCT02150967],^28,29^ and 35.5% (38/107) with a mPFS of 6.9 months in patients treated with pemigatinib (Fight-202) [ClinicalTrials.gov identifier: NCT02924376].^22^ A phase I/II study with derazantinib, limited to 29 patients, reached an ORR of 21% (6/29) with a mPFS of 5.7 months.^30,31^ Disease control rates were highly comparable across all trials (82–83%). Survival data of these trials are still immature and must be interpreted with caution due to their single-arm design and the unknown prognostic impact of FGFR2 fusions in the second line setting in biliary tract cancers. Of note, no responses were seen in iCCA patients with non-fusion FGFR2 alterations (i.e., mutations/amplifications), but disease stabilization with a mPFS comparable with that of patients with FGFR2 fusions of 6.7 months was observed in some patients with FGFR2 mutations treated with derazantinib (4/6). An additional phase II study, the FUZE trial, has recently finished recruitment, and results are pending. In this basket trial, the FGFR1-3 inhibitor debio 1347 was not only administered to iCCA patients with FGFR2 fusions, but also to patients with other solid cancers with FGFR1-3-fusion/re-arrangements [ClinicalTrials.gov identifier: NCT03834220].^32^ In a recently published phase I study with debio 1347, RECIST responses were seen across tumor types and mechanisms of FGFR activation, that is, FGFR1-3 amplification, mutation and fusions.^33–35^ Currently, two FGFR inhibitors have gained FDA approval: pemigatinib was approved in April 2020 for the treatment of advanced iCCA, whereas erdafitinib was approved for therapy-resistant urothelial cancers harboring FGFR2/3 genetic alterations.^36^ In CCA, a phase I as well as a phase IIa study in Asian patients showed promising results for erdafitinib, but due to the low patient numbers the clinical data available thus far cannot be considered fully conclusive yet [ClinicalTrials.gov identifier: NCT01703481] and [ClinicalTrials.gov identifier: NCT02699606].^37–39^ Clinical trial data on FGFR inhibitors in iCCA are summarized in Table 1.

**Table 1.** Clinical trial data on FGFR inhibitors.

Compound	Clinical trial	Study population	ORR (95% CI)	DCR (95% CI)	mDOR, months (95% CI)	mPFS, months (95% CI)	mOS, months (95% CI)	Current development	Reference
**Pemigatinib** Selective FGFR1-3 inhibitor	FIGHT-202 phase II [ClinicalTrials.gov identifier: NCT02924376]	Pre-treated A: FGFR2 fusions/rearrangements: 107 B: other FGF/FGFR alterations: 20 C: no FGF/FGFR alterations: 18	Cohort A: 35.5% (26.50–45.35) CR: 2.8% PR: 32.7% Cohort B: 0% Cohort C: 0%	Cohort A: 82% (74–89) Cohort B: 40% (19–64) Cohort C: 22% (6–48)	Cohort A: 7.5 (5.7–14.5)	Cohort A: 6.9 (6.2–9.6) Cohort B: 2.1 (1.2–4.9) Cohort C: 1.7 (1.3–1.8)	Cohort A: 21.1 (14.8– NE) Cohort B: 6.7 (2.1–16.6) Cohort C: 4.0 (2.3–6.5)	Phase III, 1st line, against Gemcitabine/Cisplatin (Fight-302) [ClinicalTrials.gov identifier: NCT03656536] recruiting	Abou-Alfa *et al.*^22^
**Infigratinib** **(BGJ 398)** ATP competitive FGFR1-3 inhibitor	Phase II [ClinicalTrials.gov identifier: NCT02150967]	Pre-treated FGFR2 fusions: 71 FGFR2 mutations: 8 FGFR2 amplifications: 3	Fusions: 31% (20.5–43.1) Mutations: 0% Amplifications: 0%	Fusions: 83.6% (72.5–91.5)	5.4 (3.7– 7.4)	6.8 (5.3–7.6)	12.5 (9.9–16.6)	Phase III, 1st line against Gemcitabine/Cisplatin (PROOF) [ClinicalTrials.gov identifier: NCT03773302] recruiting	Javle *et al.*^28^; Javle *et al.*^29^
**Derazantinib** (ARQ 087) Multi-kinase inhibitor with potent pan-FGFR activity	Phase I/II [ClinicalTrials.gov identifier: NCT01752920]	FGFR2 fusions: 29 (27 pre-treated) FGFR2 Mutations: 6 FGFR wildtype: 9	Fusions: 20.7% Mutations: 0% Wildtype: 0%	Fusions: 82.8% Mutations: 67% Wildtype: 0%		Fusions: 5.7 (4.04–9.2) Mutations: 6.7 (1.0–14.7) Wildtype: 1.4 (0.7–NA)		Phase II FGFR2 fusions, mutations and amplifications, pretreated (FIDES-01) [ClinicalTrials.gov identifier: NCT03230318], recruiting	Mazzaferro *et al.*^30^; Busset *et al.*^31^
**Debio1347** Selective FGFR1-3 inhibitor	Phase I [ClinicalTrials.gov identifier: NCT1948297]	iCCA cohort: 9 patients FGFR2 translocations: 5 FGFR1 translocation:1 FGFR2 mutation: 1 FGFR2 activating deletion:1 FGFR3 mutation: 1	iCCA cohort: PR 22% (2/9, 1 FGFR2 fusion, 1 FGFR2 activating deletion)	iCCA cohort: 66%		iCCA cohort: median time on treatment: 24 weeks (4–57 weeks)		Phase II, basket design, FGFR1-3 fusion positive solid malignancies, pretreated (FUZE) [ClinicalTrials.gov identifier: NCT03834220]	Voss *et al.*^33^; Ng *et al.*^35^
**Futibatinib** (TAS 120) Selective irreversible FGFR1-4 inhibitor	Phase I [ClinicalTrials.gov identifier: NCT02052778]	Total: 45 pretreated CCA (41 iCCA) FGFR2 fusions: 28 Other FGF/FGFR aberrations: 17 Prior FGFR-inhibitor therapy: 13	Fusions: 25% (7/28) Other: cPR: 17.6% (3/17) Prior FGFR –inhibitors: cPR: 30.7% (4/13) (3 with FGFR2 fusions, 1 FGFR2 amplification)	Fusions: 79%		Median treatment time: FGFR2 fusions: 7.4 (4.8–NC) Other: 6.8 (1.9–NC)		Phase III, 1st line against Gemcitabine/Cisplatin (FOENIX-CCA3) [ClinicalTrials.gov identifier: NCT04093362] Not yet recruiting	Meric-Bernstam *et al.*^40^; Tran *et al.*^41^
**Erdafitinib** (JNJ-42756493) Pan FGFR inhibitor	Phase I [ClinicalTrials.gov identifier: NCT01703481]	pretreated CCA subgroup with FGFR aberrations: 11 Fusions: 8/11	CCA cohort: 3/11, all PR (27.3%; 6–61)	CCA cohort: 55%	CCA cohort: 12.9 All patients: 9	CCA cohort: 5.1 (1.6–16.4) All patients: 2.9			Bahleda *et al.*^37^; Soria *et al.*^39^
**Erdafitinib** (JNJ-42756493) Pan FGFR inhibitor	LUC2001 phase IIa [ClinicalTrials.gov identifier: NCT02699606]	Only Asian patients Pretreated CCA with FGFR alterations FGFR2 fusions: 8 FGFR2 mutations: 3 FGFR3 fusions: 1 FGFR3 mutations:2	12 evaluable: PR: 50% (6/12) FGFR2 alterations: 10 evaluable ORR: 60% (6/10)	83.3% (10/12) FGFR2 alterations: 10 evaluable 100% (10/10)	6.83 (3.65–12.16)	Treatment duration: 4.83 (0.5–20.3) mPFS 5.59 (1.87–13.67) FGFR2 alterations: 10 evaluable mPFS 12.35 (3.15–19.38)			Park *et al.*^38^

CCA, cholangiocarcinoma; iCCA, intrahepatic CCA; CI, confidence interval; DCR, ; FGFR, fibroblast growth factor receptor; mDOR, median duration of response; mOS, median overall survival; mPFS, median progression-free survival; ORR, overall response rate; DCR, disease control rate.

FGFR-inhibitor associated toxicity profiles are comparable between the compounds and appear to be overall manageable, although dose reductions or interruptions are frequent (~ 60%). The most common adverse event (AE) reported across all trials was hyperphosphatemia due to the physiological involvement of the FGF23/FGFR signaling axis in phosphate homeostasis. Further frequent AEs included fatigue, alopecia, GI-toxicity (diarrhea or constipation), nail toxicities (onychodystrophy and nail loss), as well as stomatitis and dry eye.

The positive results from the already completed phase II trials can legitimately be considered a therapeutic breakthrough in a cancer with such limited treatment options, especially in second- or higher lines of therapy. Currently, three randomized controlled phase III trials are recruiting patients with FGFR2-fusions/re-arrangements that compare standard of care (gemcitabine + cisplatin) with infigratinib (PROOF) [ClinicalTrials.gov identifier: NCT03773302],^42^ pemigatinib (Fight-302) [ClinicalTrials.gov identifier: NCT03656536],^43^ or futibatinib (FOENIX-CCA3) [ClinicalTrials.gov identifier: NCT04093362],^44^ in first-line setting with the designated primary endpoint progression-free survival (PFS). Despite the promising data from the phase II studies in pre-treated patients, the trial designs nevertheless appear ambitious: a positive outcome would require that the targeted agents, which previously achieved a mPFS of 6.9 months (pemigatinib, 95% CI 6.2–9.6) or 6.8 months (infigratinib, 95% CI 5.3–7.6) in second or higher line, outperform the mPFS of 8 months reached under gemcitabine and cisplatin in the first line (ABC02 trial)^3^ in a head-to-head comparison. However, in contrast to previous “all comer” trials, these studies will recruit a genetically more homogenous group of exclusively iCCA patients, and will help to determine the prognostic and predictive value of FGFR2 fusions in biliary tract cancer. Of note, the presence of FGFR2 fusions might not only be of value as a positive predictive biomarker for FGFR-inhibitors, but may also serve as a negative predictive indicator for the use of chemotherapy.

## Primary and secondary resistance to FGFR2 directed therapies

Critical assessment of the existing data reveals that only a subset of patients with FGFR2 fusions achieves a clinically meaningful response. This observation indicates that the presence of a fusion does not necessarily guarantee sensitivity to targeted inhibitors, and points towards the existence of strong molecular networks that are capable of conferring primary resistance. Conveniently, a pre-requisite for trial inclusion was (and is) the genetic proof of the FGFR2 chromosomal alteration, which is usually conducted by performing extended panel diagnostics (e.g., *via* the FoundationOne^®^ CDx panel in the FIGHT Study). The availability of such data is highly advantageous because it accelerates the clinical annotation of therapy-relevant cause-effect relationships on the basis of the co-mutational spectrum. Initial analyses from the FIGHT-202 study already revealed that the mutational landscape of patients with FGFR2 fusion differs from patients without fusions. BAP1 alterations were enriched in fusion positive patients (38.7% *versus* 8.2%), whereas all other recurrent alterations occurred less frequently in patients with FGFR2 fusions, including TP53 as well as oncogenic drivers such as KRAS, and ERBB2.^21^ Notably, FGFR2 and IDH1 mutations were not mutually exclusive (5/107; 5.1%) raising the question: which of both druggable alterations is the main driver and should be targeted first. In respect to the question of to what extent the co-mutational spectrum affects the efficacy of FGFR2 inhibitors, initial genetic subgroup analysis of patients that received pemigatinib in the FIGHT-202 trial suggest a negative predictive value of *TP53* mutations; no responses were observed in patients with p53 mutations (zero of nine) and mPFS was significantly shorter in p53 mutant patients compared with p53 wildtype patients (*p* = 0.0003). With the increasing availability of genetic and clinical patient data from clinical trials, as well as real-life data, such integrative analysis will shed light on the molecular underpinnings of primary resistance, and will ultimately help to improve up-front patient stratification.

Beyond the observation that only one out of three patients responds to the targeted inhibitors, the long-term benefit is frequently limited, and the longest median duration of response (mDOR) in a phase II setting was 7.5 months for pemigatinib. Paralleling findings in other solid malignancies, both on- and off-target resistance can emerge under the continuous selective pressure of the tyrosine-kinase inhibitors. On-target resistance to FGFR inhibitors is defined as resistance despite the continued reliance on FGFR-fusion signaling. Generally, on target resistance results from *de novo* mutations within the FGFR2-kinase domain of the chimeric protein that interfere with the binding of the small molecule inhibitor. Treatment with a second FGFR-targeted inhibitor can be considered in some cases. However, the complexity of secondary resistance mutations is highlighted by initial data from liquid biopsies that confirm the frequent presence of not only a single but multiple different mutations in the FGFR2 kinase domain.^45,46^ Investigations using pre-clinical models provided convincing evidence that FGFR inhibitors exhibit distinct activity profiles against secondary FGFR mutations, thus indicating that the genetic alterations can (and, in the future, should) guide selection of the most appropriate compound. For instance, the irreversible FGFR inhibitor futibatinib (TAS-120), soon be entering a phase III trial against gemcitabine and cisplatin in first line in FGFR2 gene rearranged iCCA (FOENIX-CCA3), may not only be an option for first-line treatment but also as rescue treatment, because it remains active against a subset of secondary mutations that may emerge under prior treatment with ATP-competitive inhibitors, such as infigratinib or debio 1347. The translational significance of these findings has been clinically confirmed in a subset of patients that progressed under FGFR-inhibitor therapy, but responded to an FGFR inhibitor re-challenge with futibatinib.^40,41,46^

The Achilles heel of most FGFR inhibitors is that their activity depends on binding to the ATP binding pocket of the tyrosine kinase. So-called gatekeeper mutations frequently affect residue V564 (V565 annotated according to FGFR2 isoform IIIb), and can inhibit the drug from accessing the hydrophobic pocket due to steric hindrance. Futibatinib appears to retain limited potency against selected mutations at the gatekeeper residue, such as V565I, but not V565F, which was still relatively sensitive towards debio 1347 in an *in vitro* assay.^46^ The most promising activity profile in that regard, however, has been attributed to a compound that was developed as an ATP-competitive pan-FGFR inhibitor, LY2874455. Wu *et al*. provided *in vitro* evidence that LY2874455 has the potential to overcome drug resistance driven by FGFR gatekeeper mutations.^47^ However, although LY2874455 demonstrated good tolerability in patients with solid organ malignancies,^48^ the clinical development of LY2874455 has been discontinued.

In the near future, longitudinal liquid biopsy diagnostics starting prior to the initiation of targeted therapies will likely become an important tool to track the evolution of secondary resistance mutations that mediate treatment failure and to guide selection of adequate second line therapy. Of note, not all NGS panels used for the diagnosis of FGFR2 fusions necessarily cover all the genomic regions coding for the secondary resistance mutations.

Another opponent of long-lasting responses to FGFR inhibitors is off target resistance. Off target resistance bypasses oncogene addiction through the acquisition of novel (epi-) genomic alterations that converge on the activation of alternative pathways. Specifically, in CCA patients under treatment with FGFR inhibitors, the PI3K/AKT pathway has been reported to convey secondary resistance.^46^ This is in line with pre-clinical data from other tumor entities that harbored genetic FGFR1, FGFR2 or FGFR3 alterations, and developed AKT-mediated resistance after initially responding to FGFR inhibitors.^49–51^ It is well conceivable that a considerable overlap exists between mechanisms that lead to off-target resistance and those that cause primary resistance. A more global understanding of mechanisms that convey off-target resistance will aid in the identification of viable co-treatment strategies that delay time to progression or re-establish disease control.

## Outlook: FGFR-directed combination therapies

After completion of the currently recruiting trials that address the role of FGFR-targeted monotherapy, the field will likely move towards combination approaches. One potential future concept that is currently under discussion will be the combination of immune-oncology (IO) and FGFR inhibition. Highly promising results for the combination of IO and targeted therapies have already been reported for other solid malignancies, such as in ERBB2/HER-2 positive gastric cancer [ClinicalTrials.gov identifier: NCT02954536].^52^ Thus far, only pre-clinical data exist for dual targeting of the FGF receptors and immune checkpoints. Initial data in murine model systems implicate that FGFR-inhibition can alter the immune microenvironment of tumors and enhance the anti-tumor T-cell responses.^53^ In addition, some FGFR inhibitory compounds also exhibit activity against other receptor tyrosine kinases: for instance, derazantinib inhibits the *Colony Stimulating Factor 1 Receptor (CSF1R) in vitro* at similar concentrations as required for the inhibition of FGF-receptors. Tumor macrophage modulation through CSF1R blockade may render tumors more responsive to T-cell checkpoint inhibition.^54,55^

## Summary

During the last 4 years, the portfolio of available FGFR-inhibitory compounds quickly expanded. The growing interest in FGFR inhibitors as targeted therapy for FGFR2-fusion positive iCCA is fueled by exceptionally encouraging results from clinical phase II trials in pre-treated patients, which resulted in the recent United States Food and Drug Administration (FDA) approval of pemigatinib for the treatment of advanced iCCA patients with FGFR2 fusions. Ongoing phase III trials are comparing the efficacy of the targeted agents with gemcitabine and cisplatin in the first therapeutic line.

The number of identified possible FGFR2 fusion partners is steadily growing and currently already exceeds 150 different genes. Therefore, a diagnostic approach that is “unbiased” with regards to the fusion partner is crucial for the reliable identification of FGFR2 fusion positive patients, and to ensure that effective treatment strategies are not withheld from this genetically defined patient cohort.

In the future, a better understanding of the genetic and molecular alterations that influence therapy response will be important to improve patient selection and optimize therapeutic outcome. In this regard, a meta-analysis of clinical and matched genomic data from recent and ongoing trials would likely be highly informative.

In patients that are under treatment with targeted therapies, probably the most daunting challenge is the development of secondary resistance. Here, we face both a diagnostic as well as a therapeutic dilemma: a tissue biopsy from a single site might not be representative regarding the multiplicity of on- and off-target resistance mechanisms. Liquid biopsy diagnostics as a non-invasive strategy has the potential to become a powerful tool to monitor and to better understand the evolution of resistance.

Considering the recent approval of pemigatinib and the ongoing clinical trials with FGFR inhibitors in iCCA, it appears mandatory to start planning ahead and to conceive early strategies that will help to exploit the full potential of FGFR as a target for precision oncology. A close collaboration between clinical experts and basic scientists will be of utmost importance to understand the molecular underpinnings of therapeutic failure, to navigate the design of optimized compounds, and to develop experimentally informed co-treatment as well as sequential-treatment strategies.
